# Reverse adoption of information and communication technology among organisers of academic conferences

**DOI:** 10.1007/s11192-022-04616-y

**Published:** 2023-02-19

**Authors:** Martin Thomas Falk, Eva Hagsten

**Affiliations:** Department of Business and IT, School of Business, University of South-Eastern Norway, Campus Bø, Gullbringvegen 36, 3800 Bø, Norway

**Keywords:** Academic conferences, Hybrid conferences, Virtual conferences, Travel restrictions, Planning horizon, Multinomial logit model

## Abstract

This study examines the formats offered for academic conferences in the mature stages of the COVID-19 pandemic. Two out of three organisers discontinue their usage of online video tools and focus on in-person conferences. Only one out of five conferences offers hybrid solutions and even fewer a virtual alternative (13%). Data for the analysis originate from 547 calls for proposals announced in Spring 2022 for conferences to be held during the period August 2022 to July 2023. Estimates using a multinomial logit model show that the planning time is significantly related to the choice of format offered. The longer the lead time, the more likely it is to offer an in-person conference. International travel restrictions and bans on gatherings for the location of the venue at the time of planning are significantly related to the choice of virtual, but not hybrid formats. There are also large differences in the choice across disciplines, with conferences in arts and humanities as well as natural sciences showing the lowest preference for the virtual format.

## Introduction

Faced with the alternative of cancelling or postponing the academic conference, many organisers chose to change their format to virtual in the early phases of the COVID-19 pandemic (Mubin et al., 2021; Roos et al., 2020; Wu et al., 2022). Fast improvements in videoconferencing technologies drastically alter the way academic conferences can be organised and participants are generally quick to learn how to use the software and tools. Examples include sharing of presentations, codes and data, use of the chat functions, smooth transition between sessions and recording of sessions (Skiles et al. 2021; Wu et al., 2022). This means that the traditional face-to-face setting is no longer the only possible format for an academic conference. The rapid increase in the use of video conference tools could be identified as a technological innovation or application that is diffused through society (Rogers, 2003).

Virtual conferences have several advantages over face-to-face conferences. They allow many participants, are easily accessible independent of where the researchers are based (Estien et al., 2021; Roos et al., 2020; Wu et al., 2022; Yates et al., 2022), are environmentally friendly (Leochico et al., 2021; Medina & Shrum, 2022; Tao et al., 2021; Tseng et al., 2022; van Ewijk & Hoekman, 2021) and facilitate the attendance of underrepresented or less mobile groups (Estien et al., 2021; Fraser et al., 2017; Skiles et al., 2022; van Ewijk & Hoekman, 2021; Wu et al., 2022). There are, however, also disadvantages with virtual conferences such as exhaustion or Zoom fatigue (Bennett et al., 2021; Fosslien & Duffy, 2020; Hacker et al., 2020), lack of interaction (Roos et al., 2020) as well as different time zones and technical shortcomings (Tseng et al., 2022).

The purpose of this study is to examine factors that influence the choice of format for a proposed academic conference in the mature stages of the COVID-19 pandemic, distinguishing between in-person, hybrid and virtual alternatives. A multinomial logit model is employed that allows simultaneous estimations of the probability of offering a specific conference format. Explanatory variables include the field of expertise, constraints on meetings and international travel as well as the lead time of the planned conference. For this purpose, data from 547 academic conference calls for papers open in March 2022 are compiled. These conferences are announced for a period starting in August 2022 and ending in July 2023.

There is a vast learning effect for the participants and organisers of virtual scientific conferences in the wake of the COVID-19 pandemic. Tseng et al. (2022) point out that this experience creates a good opportunity for scientists and institutions to re-think the need for face-to-face conferences and Gifford (2022) concludes that it is an ethical dilemma with academic conference participants flying around to share their research findings (which is often related to climate change adaptation), while the travel itself contributes to global climate change. Some researchers even believe that virtual academic conferences are the new norm (Foramitti et al., 2021; Gill, 2021; Roos et al., 2020). Although virtual conferences may not completely replace face-to-face meetings, several studies demonstrate that this format is becoming an accepted alternative (Nahai, 2021; Roos et al., 2020). Recent literature also stresses the benefits of hybrid conferences since they both offer the participants larger flexibility and would also to some extent decarbonise conference travel (Puccinelli et al., 2022; Wu et al., 2022).

Early studies of virtual academic conferences focus on the choice of format available during the acute initial phases of the COVID-19 pandemic rather than what is actually preferred in the longer run (Falk & Hagsten, 2021; Mubin et al., 2021). The experience and satisfaction of participants of virtual or hybrid conferences and their advantages and disadvantages are also discussed (Etzion et al., 2022; Hohlfeld et al., 2021; Medina & Shrum, 2022; Niner & Wassermann, 2021; Puccinelli et al., 2022; Raby & Madden, 2021a; Roos et al., 2020; Yates et al., 2022). Additional literature compares the number of participants and their profiles between virtual and physical conferences (Skiles et al., 2022; Wu et al., 2022). Another strand of research emphasises the carbon emission reduction of online and hybrid conferences (Skiles et al., 2022) or undertakes a life-cycle assessment of different formats (Duane et al., 2021; Tao et al., 2021).

Several studies suggest that little attention is paid to the possible discontinuation of the usage of an innovation in the information system life cycle (Furneaux & Wade, 2011; Maier et al., 2015). Exceptions include individuals who quit social media for a period of time (Cao & Sun, 2018; Maier et al., 2015; Ng, 2020; Zhang et al., 2016) or mobile shoppers (Chen et al., 2019). This study contributes empirical evidence on the direction of formats offered for academic conferences in the medium run from the horizon of a mature phase of the COVID-19 pandemic. Hybrid conferences are relatively new and their relevance is not yet thoroughly analysed.

The remaining study is structured as follows: “The conceptual background” section provides the conceptual background and the methodological approach is outlined in “The empirical model” section. Data are presented in “The data and descriptive statistics” section while “The empirical results and discussion” section encompasses a discussion and presentation of results. “The conclusions” section concludes the analysis.

## The conceptual background

In this section, a description of typical formats for academic conferences as well as literature on their advantages and disadvantages leads to a dissection of the purpose into four hypotheses.

### Alternative conference formats

Based on a systematic literature review, Leochico et al. (2021) identify three general ways of conducting scientific conferences: face-to-face (in-person); online (virtual, digital, remote, webinar); and hybrid (mix of in-person and virtual). Before the COVID-19 pandemic, face-to-face is the most common conference format, which means that participants travel to the venue and consequently also generate carbon dioxide (for an exception of a virtual conference in 2019, see Abbott, 2020). During the initial phases of the COVID-19 pandemic, many academic conference organisers face the opportunities to postpone, cancel or change to a virtual format (Mubin et al., 2021). Online conferences can take place either synchronously (live/real-time) or asynchronously (recorded) or as a mix of these two kinds of sessions (Boluk et al., 2022). Well into the more mature phases of the pandemic (mid-2021), the hybrid format appears as an alternative (Puccinelli et al., 2022).

The distinction between “in-person”, “hybrid” and “virtual” may in principle be too broad since there are now many new conference formats. One alternative is the multiple conference site approach with virtually networked and remote (sub-regional or local) centres enabling face-to-face attendance (Counsell et al., 2020; Klöwer et al., 2020; Leochico et al., 2021; Sarabipour et al., 2021; Skiles et al. 2021; Tao et al., 2021; van Ewijk & Hoekman, 2021). This means that one conference can take place simultaneously in different, virtually connected centres, so that participants only need to travel to their nearest hub to interact with a certain group of scientists (Klöwer et al., 2020; Sarabipour et al., 2021; Tao et al., 2021). This kind of approach can be called “the hub-and-spoke conference model”, “a conference within a conference” or “the hybrid hub conference” (Leochico et al., 2021; Skiles et al., 2022). Klöwer et al. (2020) propose a three-hub locations model to reduce the number of air miles travelled by academics. Adding more hubs may reduce the carbon footprint by 60–70%, while virtual participation is kept at less than 50% (Tao et al., 2021). Thus, recent literature reveals that there is possibly more alternative conference format available now than before the pandemic and they tend to be less distinct.

Despite the increasing literature on the need for decarbonisation of academic conferences only one out of 547 calls for papers offer a virtual multi-hub model (one onsite hub in Europe, one in North America and one online in South America) (Table 1) (source: communicatingsustainability2022.com/conference-programmes). This conference format is so rare that it cannot be included in the modelling.

**Table 1:** Examples of conference formats offered

Name	Location	Date	Conference modes
ICTE3	Ho Chi Minh, Viet Nam	Dec 3, 2022	The Conference will be hybrid, virtual mode for international delegates and offline for local delegates
SEAC 2022	Oxford, MS, United States	Oct 6, 2022	In-person conference with limited slots for virtual presentation
CS2022	Glasgow, UK	Sep 6, 2022	The conference will take place in person at the University of Glasgow, at Goucher College, and online at the Federal University of Sergipe
CIST2022	Indianapolis, IN	Oct 15, 2022	Attendees can participate in-person or remotely. Presenters should plan to attend in person. However, exceptions can be made for those who are unable to travel
PPAM 2022	Gdansk, Poland	Sep 11, 2022	We strongly encourage submissions from people who plan to attend the conference in person. For those who cannot travel, we will offer the possibility of remote participation
FinanceCom 2022	Enschede, Netherlands	Aug 23, 2022	We aim for an on-site event, while hybrid and online participation will be facilitated
32nd SOFT	Dubrovnik, Croatia	Sep 18, 2022	On-site registration limited to 500 participants
ICITR2022	Moratuwa, Sri Lanka	Dec 7, 2022	Due to the COVID-19 pandemic, ICITR 2022 will be held as an ONLINE EVENT, with limited physical attendance
CIDA International 2022	Ankara, Turkey	Oct 12, 2022	Online presentations can be made but face-to-face symposium is prioritized
ICICM 2022	Xi’an, China	Oct 28, 2022	Only listener can be allowed to register online
KNOWCON 2022	Olomouc, Czechia	Dec 8, 2022	Passive online-participation of BA, MA and PhD students is free
OSF2022NL	Amsterdam, Netherlands	Sep 1, 2022	Sessions and workshops will be held in-person only; we intend to livestream several plenary sessions
ESDiT2022	Oegstgeest, Netherlands	Oct 6, 2022	Keynote addresses will be hybrid, in-person and virtual, so as to provide worldwide access to topics of the conference
CIRS 2022	Malabe, Sri Lanka	Nov 10, 2022	Conference will be held in the Auditorium of CINEC Campus with Live Streaming on Facebook and YouTube

*Source* Easychair

The statements in the conference proposals show that the preferred (or possible) conference format is not entirely clear. Some organisers express their preference for in-person participation but keep the conference open for online presentations under specific conditions when, for instance, travel is not possible (“limited time for virtual conferences”, “presenters should be present in-person”, “face-to-face presentations have priority”). There are also conferences that offer passive online participation without presentation. In addition, the calls reveal a certain degree of ambiguity from the perspective of the conference organisers. One conference organiser, for instance, asks participants to indicate their preferences for the format of the event when they submit their papers (https://easychair.org/cfp/Anticipation22).

Although the distinction among the different categories is not entirely clear and the boundaries are somewhat blurred, the following three categories are used in the empirical part: (i) in-person conference only, (ii) hybrid conference of all forms including passive participation of virtual conferences and (iii) virtual conference only with no in-person participation.

There are two main motivations among organisers for offering a hybrid or virtual conference. One is the actual COVID-19 restrictions in some parts of the world, the other is environmental reasons. Very few conference organisers mention the possibility of virtual participation in order to reduce the carbon emissions generated by air travel to the conference location. An exception is the “audiomostly” conference where online participation is mentioned because it is more environmentally friendly. This is described in the conference call as: “To endorse ecological green “science aspects “ a special online paper session will be provided for presenters who cannot travel to Austria” (https://easychair.org/cfp/am22, https://audiomostly.com/2022/).

### Advantages and disadvantages of conference formats and research hypotheses

Several studies document that the advantages of online conferences include lower travel costs, reduced organisation costs and removal of geographical and administrative barriers (Roos et al., 2020; Wu et al., 2022). Literature also agrees that replacing or supplementing face-to-face conferences with virtual ones would help make them more accessible and reduce their carbon footprint (Etzion et al., 2022; Roos et al., 2020; Wu et al., 2022).

Based on a review of the literature, Leochico et al. (2021) conclude that not only online but also hybrid conferences have several advantages, such as a significant reduction in carbon dioxide emissions and various forms of waste (time, money, resources, energy). Experiences based on fully online conferences show that they attract larger groups of attendees, even those who during other circumstances would not have been able to participate (Niner & Wassermann, 2021; Raby & Madden, 2021b; Skiles et al., 2022; Wu et al., 2022). Medina and Shrum (2022) note that researchers value the larger number of participants, greater geographic and occupational diversity, reduced carbon emissions, lower travel and transport costs, and ease of viewing the lectures later (through recordings).

Skiles et al. (2022) use a survey of participants and find several benefits of online conferences beyond the overall larger group of participants; women, historically underrepresented institutions, students and postdoctoral researchers. Besides inclusivity and diversity, online conferences may also overcome geographical, cultural, resource and disability barriers (Leochico et al., 2021). Apart from their positive environmental impact and the higher inclusiveness, online scientific conferences offer a variety of benefits such as large savings in time, costs (predictable and unpredictable) and positive effects on the health status (e.g. reduced risk of COVID-19 infection, jet lag, insomnia, noise, social strain, work-related stressors) (Leochico et al., 2021). In spite of the many presumptive advantages for different groups of scholars and the environment, few of the conference organisers included in present analysis state specifically such concerns in their calls.

There are also apparent disadvantages of online conferences. Several studies document the difficulty of networking as the main problem, which affects the strengthening of academic collaboration and the consolidation of careers (Medina & Shrum, 2022; Roos et al., 2020). Other studies argue that online can never be a substitute for physical conferences since it faces certain obstacles that cannot be overcome, including distraction/multitasking of participants or learners and impaired social interactions (Fraser et al., 2017; Leochico et al., 2021; Roos et al., 2020). Issues with different time zones can only partly be solved by longer time windows for the conference (Tseng et al., 2022) The underlying technology, such as the speed of the broadband at the host or participant location as well as stress originating from the online format, may also be crucial aspects of importance (Anh et al., 2022; Falk & Hagsten, 2021). Sarabipour et al. (2021) believe that the right incentives can help to overcome some of the disadvantages of online conferences. This could encompass hybrid, local hubs inclusiveness for early careers scholars and those with difficulties to travel.

Thus, given the advantages and disadvantages, organisers can be expected to choose the format that is associated with the highest benefit. Such a cost–benefit analysis may tend more often towards online solutions than before, especially with its improved technology and sharply rising learning curves (Brem et al., 2021). This analysis refers to calls for papers open in March 2022, two-years into virtual conferencing for most organisers and participants. At this stage there might be an accumulated need to find the “new normal”. According to the conference calls, this new normal has a strong resemblance to the pre-pandemic behaviour, implying a discontinuation of an innovation or technological application despite the fact it has high quality and is spread through its whole society. In other cases, the use of a technology is commonly discontinued because of the need for replacement, the innovation has become obsolete or too expensive (Rogers, 2003). Other relevant factors are technostress (Ng, 2020) or information overload (Cao & Sun, 2018; Zhang et al., 2016).

Based on information from the calls for papers and indications in literature that the advantages of virtual conferences may not yet outweigh face-to-face formats, the first and second hypotheses are formulated:

#### H1

A relaxation of travel (mobility) and gathering restrictions increases the number of in-person conferences offered.

#### H2

A longer planning horizon in the mature phase of the COVID-19 pandemic increases the number of in-person conferences offered.

Environmental concerns, costs and inclusion issues are aspects of importance known to conference organisers already before the pandemic. Together with the increased quality of virtual conference tools and the improved skills in operating them, it is possible that these concerns are given higher priority than before, leading to the third and fourth hypotheses:

#### H3

The number of in-person conferences offered varies across academic fields.

#### H4

There is an increased interest in offering hybrid conferences.

## The empirical model

The choice of format for an academic conference is investigated by the use of a multinomial logit model. This model allows a simultaneous assessment of the likelihood of proposing a certain conference format. The proposed format can be motivated by a random utility model where the maximum expected benefit of the organiser and the conference participants depends on the various offerings. Independent variables include the planning horizon (or lead time), academic discipline and the presence of COVID-19 restrictions at the time of the planning. Travel restrictions are in place in several countries during the planning phases. These include limitations on the number of participants and events, leading to cancellation of conferences and trade fairs as well as increased use of digital meetings (Hale et al., 2020, 2021).

The standard multinomial model can be expressed as (Greene, 2020):

$${\pi }_{ij}=\mathit{Pr}({Y}_{i}=j|{X}_{i})=\frac{{e}^{{X}_{i}{\beta }_{j}}}{{\sum }_{k=1}^{J}{e}^{{X}_{i}{\beta }_{k}}}$$

where $\mathit{Pr}({Y}_{i}=j|{X}_{i})$ is the probability that the call for papers *i* has the format *j* (= *1,…,J)* given a column vector of exogenous explanatory variables *X*_*i *_for that observation.

The multinomial logit model pairs each response alternative with an arbitrary baseline category. In this analysis, there are three possible responses (J = 3): Pf for an in-person conference format (j = 1), Ph for a hybrid or with virtual elements integrated (j = 2) and Pv for a virtual conference (j = 3). Face-to-face conferences are, as the largest group, set as the reference category. The conference organiser chooses the format *j* if its utility is largest for this type, that is, $U_{itj} = \mathop {\max }\limits_{1 \le k \le J}^{{}} \{ U_{itk} \} ,j = 1,...,J$, given the following utility function:

$${U}_{ij}={X}_{i}{\beta }_{j}+{e}_{ij}$$

This equation contains the observable component ${X}_{i}$ and the random error term *e*_*ij*_ leading to the specific multinomial logit model:

$$log\left( {\frac{{\pi _{{ij}} }}{{\pi _{{i3}} }}} \right) = X_{i} ^{\prime } \beta _{j} = \beta _{0} + \sum\limits_{{RT = 1}}^{3} {\beta _{{1RT}} Restrictionstravel_{i}^{{RT}} } + \sum\limits_{{RG = 1}}^{3} {\beta _{{2RG}} Restrictionsgatherings_{i}^{{RG}} } + \sum\limits_{{D = 1}}^{8} {\beta _{{3D}} Discipline_{i}^{D} } + \beta _{4} Planningtime + \beta _{5} Planningtime_{i}^{2} + \varepsilon _{i} ,$$

where *j* = 1,2. The specification includes sets of dummy variables for international travel restrictions, $Restrictionstravel$, and for bans on gatherings, $Restrictionsgatherings$. $Discipline$ is a set of dummy variables for the discipline and *Planningtime* measures the planning horizon in months (5,…,6) as from March 2022 (T = 0 is 3/2022) and ${\varepsilon }_{i}$ is the error term. To allow for a non-linear relationship, a quadratic measure of time, ${Planningtime}^{2}$ is added to the specification.

The multinomial logit model is estimated by Maximum Likelihood and the standard errors are clustered at the country level to account for the fact that the error term may be correlated across conferences planned within the same country. Alternatively, the multinominal probit model can be used (Greene, 2020).

## The data and descriptive statistics

This study uses information from Easychair on 611 conference calls around the world scheduled to be held between August 2022 and July 2023 (www.easychair.com). Data are collected at the end of March 2022. A wide range of academic fields are included: Arts and Humanities, Life Sciences, Business and Management, Education, Energy, Health Sciences, Mathematics and Statistics, Physical Sciences, Social Sciences, Computer/Information, Earth and Environmental Sciences. Engineering and technology conferences are not considered because they have different characteristics (size and regular participation from industry). 

Chemistry and Genomics and Bioinformatic are also excluded from the dataset because of the small number of conferences. Conferences advertised in languages other than English are exempt (# 35) as well as are the four postponed conferences. Several studies mention so-called “questionable conferences” that offer academic meetings of low quality (Kulczycki et al., 2022; Lang et al., 2019). There are 15 announced conferences in the database that either do not provide details of their academic committees or lack a link to an academic institution. These conferences are considered questionable and excluded from the empirical analysis, leading to a final database of 547 conferences. Information about bans on gatherings and international travel restrictions for the month of March (maximum value for this month) is based on Hale et al. (2021) (Table 6, Appendix).

Most conferences are held in the field of computer science (57%) followed by social sciences (9%) (Table 2). Restrictions on gatherings and international travel are partly in use at the time of publishing the call for proposals in March 2022. This means that 17% of the conference locations have an arrival ban on all regions and only one third have no restrictions at all on large gatherings or very large gatherings (32%) (Table 3).

**Table 2:** Descriptive statistics planned conferences

	%
*Academic field*	
Arts and humanities	5.1
Educational science	3.8
Energy	4.9
Health sciences	3.8
Mathematics and statistics	6.4
Social sciences	9.0
Computer science and Information technology	56.7
Natural science	5.5
Business, management, economics	4.8
*Travel restrictions end of March 2022*	
0–1 No travel restrictions/screening	28.2
2 Arrival quarantine from some or all regions	31.1
3 Ban on arrivals from some regions	23.8
4 Ban on all regions or total border closure	17.0
*Restrictions on gatherings end of March 2022*	
0–1 On very large gatherings/no restrictions	31.8
2 On gatherings between 100–1000 persons	17.2
3 On gatherings between 10–100 persons	9.1
4 On gatherings of less than 10 persons	41.9
	Number of months (mean)
*Planning time organiser*	6.5

*Source* https://covidtracker.bsg.ox.ac.uk, Easychair, own calculations

**Table 3:** Conference formats offered by field and restriction (%)

	In-person	Hybrid	Virtual
*Academic field*			
Art and humanities	67.9	28.6	3.6
Education science	33.3	42.9	23.8
Energy	74.1	14.8	11.1
Health sciences	71.4	19.1	9.5
Mathematics Statistics	60.0	25.7	14.3
Social sciences	42.9	30.6	26.5
Computing	71.0	18.4	10.7
Natural science	56.7	36.7	6.7
Business and management	61.5	7.7	30.8
Total	65.1	21.8	13.2
*Restrictions on gatherings end of March 2022*			
0 No restrictions	65.6	27.2	7.2
1 On very large gatherings	65.3	22.5	12.2
2 On gatherings between 100–1000 persons	60.6	24.5	14.9
3 On gatherings between 10–100 persons	50.0	28.0	22.0
4 On gatherings of less than 10 persons	69.9	16.2	14.0
*Travel restrictions end of March 2022*			
0 No travel restrictions	80.0	20.0	0.0
1 Screening	67.8	23.5	8.7
2 Arrival quarantine from some or all regions	67.7	21.2	11.2
3 Ban on arrivals from some regions	63.9	20.8	15.4
4 Ban on arrivals from all regions or total border closure	57.0	21.5	21.5

*Source* https://covidtracker.bsg.ox.ac.uk, Easychair and own calculations

Descriptive statistics show that an overwhelming majority, two thirds of the conferences, are planned to be held in-person, approximately one fifth as hybrid and 13% in the virtual format (Table 3). This coincides with the observation of Wu (2022), that the virtual mode already in the mature stages of the COVID-19 pandemic is meeting a fast decline despite available technology and experience. Conferences in the field of educational science have the lowest level of planned in-person conferences for the benefit of (first) hybrid and (second) virtual formats. Even conferences planned for locations with high levels of travel or gathering bans at the time for the call are to a large extent offered as face-to-face events.

## The empirical results and discussion

Results of the multinomial logit estimations show that the choice of the conference mode relates to the academic field and to the level of restrictions on gatherings and international travel (Table 4). Several fields do not provide virtual alternatives at all. Planned conferences in educational science have the highest probability of being held in a hybrid format (*p* < 0.01) followed by conferences in social and natural science (both at *p* < 0.05). Conferences in computer science and arts as well as in humanities, on the other hand, have the lowest probability of being held in a virtual format (*p* < 0.05 each). Restrictions on gatherings between 10–100 persons have a positive sign and are highly significant (p-value < 0.01) in determining the probability of virtual conferences while a total ban on international travel is significant at the five percent level. This implies that, despite the high level of usage, quality of videoconferencing tools available and experiences achieved, the adoption is generally discontinued. Thus, Hypotheses 1, 2 and 3, relating to in turn travel restrictions, planning time and variation across disciplines, cannot be rejected.

**Table 4:** Multinomial logit estimations of conference formats offered (coefficients)

	Hybrid		Virtual	
	Base category: in-person conference			
	Coeff	z-stat	Coeff	z-stat
*Academic field*				
Arts and humanities (reference: business and management)	1.177	1.28	− 2.268**	− 2.16
Educational science	2.379***	2.88	0.306	0.47
Energy	0.554	0.59	− 1.335*	− 1.80
Health sciences	0.797	0.81	− 1.430*	− 1.92
Mathematics and statistics	1.356*	1.71	− 0.690	− 1.02
Social sciences	1.695**	2.05	0.195	0.32
Computer science and IT	0.817	1.14	− 1.226**	− 2.22
Natural science	1.680*	1.83	− 1.550*	− 1.74
*Travel restrictions*				
2 Arrival quarantine from some or all regions (reference: No restrictions/screening)	0.208	0.71	0.272	0.60
3 Ban on arrivals from some regions	0.087	0.31	0.547	1.23
4 Ban on all regions or total border closure	0.318	0.93	1.196**	1.99
*Restrictions on gatherings*				
2 On gatherings between 100–1000 persons (reference category: very large gatherings/no restrictions)	− 0.004	− 0.01	0.030	0.06
3 On gatherings between 10–100 persons	0.417	1.26	1.301***	2.68
4 On gatherings of less than 10 persons	− 0.527**	− 2.29	0.313	0.72
*Planning time organiser*				
Number of months from March 2022	1.341*	1.95	0.268	0.44
Months squared from March 2022	− 0.089*	− 1.87	− 0.022	− 0.65
Constant	− 6.894***	− 2.71	− 2.205	− 0.90
Log pseudolikelihood	− 448.397			
Pseudo *R*^2^	0.067			

The standard errors are cluster-adjusted across countries (#71). The baseline is the in-person conferences

*Source* https://covidtracker.bsg.ox.ac.uk; Easychair and own calculations

**Fig. 1:**
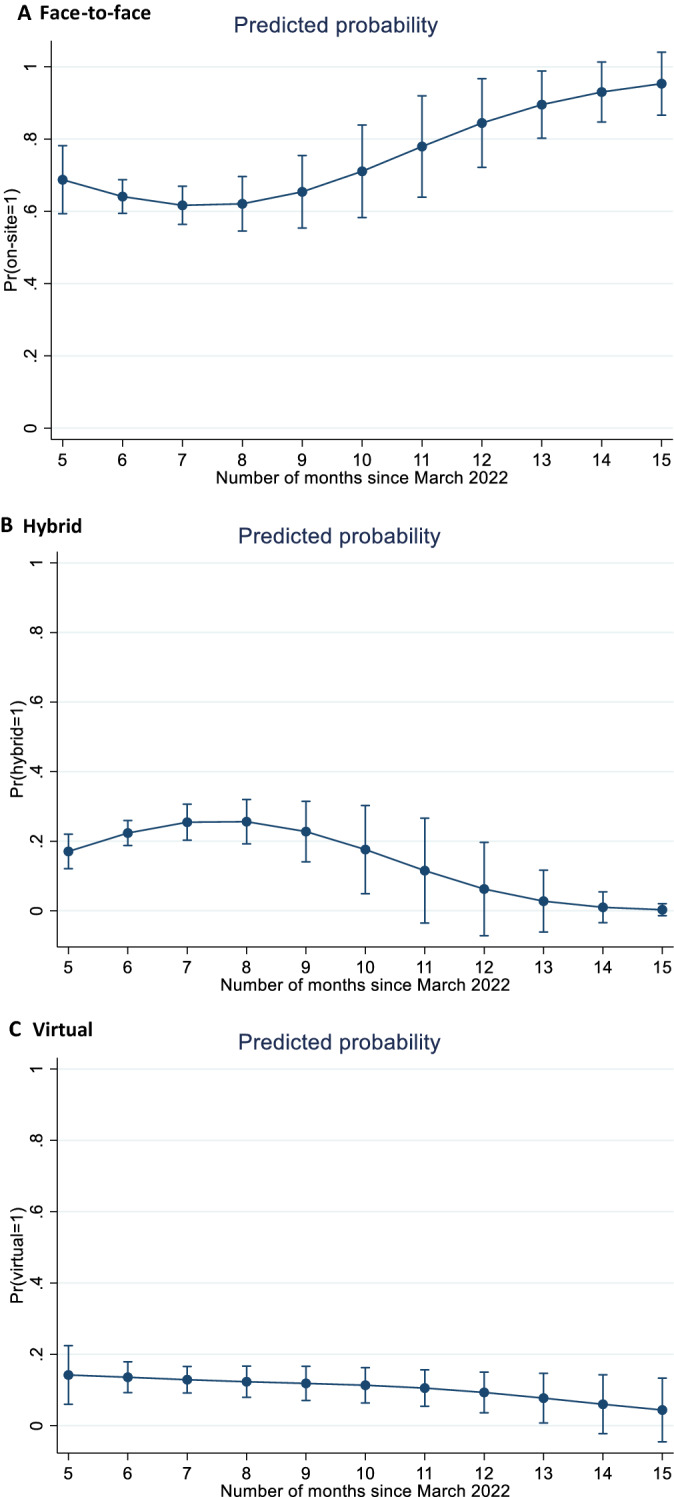
**A**–**C** Predicted probability of conference formats offered. Notes Calculated based on mean values and the coefficients in Table 4. *Source* Easychair and own calculations

By use of the Wald test, the planning time available and its squared term appear jointly significant at the five percent level. This suggests that the choice of different conference formats changes over time, in that both hybrid and virtual models are declining, and the face-to-face version is increasing (Fig. 1A–C). Following this, the statement in Hypothesis 4 of an increased interest in hybrid conferences cannot be verified. Those conferences with the longest planning horizon are predicted to appear physically to almost 100%. Although this group of conferences is relatively small, there are indications that organisers prefer the returning to the old well known conference format in the mature stages of the pandemic.

The field of educational science exhibits the weakest preference for in-person conferences (*p* < 0.01) (Table 5). The marginal effect of -0.34 indicates a 34 percentage points lower probability for this category compared with the reference category business, management and economics. Educational sciences and natural sciences are also leading in planned hybrid conferences with marginal effects of 0.37 and 0.27 (*p* < 0.01 and *p* < 0.05, respectively) followed by the field of mathematics and statistics (0.24 and *p* < 0.05). Arts and humanities as well as natural science have the lowest preference for virtual conferences with marginal effects of -0.27 and -0.21 (each < 0.05) followed by health sciences with a marginal effect of -0.17 (*p* < 0.05) and computer sciences with a marginal effect of -0.15 (*p* < 0.01) (Table 5).

**Table 5:** Multinomial logit estimations of conference formats offered (marginal effects)

Academic field	In-person		Hybrid		Virtual	
	dy/dx	z-stat	dy/dx	z-stat	dy/dx	z-stat
Arts and humanities (reference cat. business and management)	0.02	0.15	0.25	1.64	− 0.27**	− 2.31
Educational science	− 0.34***	− 2.65	0.37***	2.96	− 0.04	− 0.54
Energy	0.03	0.22	0.13	0.84	− 0.16*	− 1.81
Health sciences	0.01	0.04	0.17	1.07	− 0.17**	− 2.13
Mathematics and statistics	− 0.13	− 0.93	0.24**	1.97	− 0.11*	− 1.65
Social sciences	− 0.24*	− 1.77	0.27**	2.18	− 0.03	− 0.50
Computing	− 0.01	− 0.12	0.17	1.49	− 0.15***	− 2.58
Natural science	− 0.10	− 0.71	0.31**	2.21	− 0.21**	− 2.21
*Travel restrictions*						
2 Arrival quarantine from some or all regions (reference: No restrictions/Screening)	− 0.05	− 0.84	0.03	0.56	0.02	0.48
3 Ban on arrivals from some regions	− 0.05	− 0.98	0.00	− 0.03	0.06	1.14
4 Ban on all regions or total border closure	− 0.13*	− 1.76	0.02	0.34	0.12*	1.86
*Restrictions on gatherings*						
2 On gatherings between 100–1000 persons (reference category: very large gatherings/no restrictions)	0.00	− 0.02	0.00	− 0.02	0.00	0.07
3 On gatherings between 10–100 persons	− 0.16**	− 2.18	0.03	0.65	0.13**	2.54
4 On gatherings of less than 10 persons	0.05	0.88	− 0.09**	− 2.46	0.05	1.00
Planning time from March 2022 in months	− 0.02	− 0.92	0.03*	1.92	− 0.01	− 0.35

Asterisks *, ** and *** denote significance at the 10, 5 and 1% levels. The marginal effects are calculated based on the sample means

*Source* https://covidtracker.bsg.ox.ac.uk, Easychair and own calculations

To get an understanding of the variations, the predicted probabilities are calculated for the planning time that spans from five (August 2022) to tvelwe months  (July 2023). These results clearly reveal that there is a tendency to go back to the standard conference format (Fig. 1A–C).

Given the relatively small number of planned future conferences with virtual elements the question arises why this format seems to be abolished at the earliest possible opportunity, despite the improved technology, increased skills as well as presumptive lower costs, less discrimination against participants from certain locations and important environmental saving aspect.

Biesbroek et al. (2013) list seven categories of barriers to climate change adaptation that could also apply to the provision of conferences with virtual elements: (1) conflicting schedules, (2) substantive, strategic and institutional uncertainty, (3) institutional overcrowding and emptiness, (4) institutional fragmentation, (5) lack of awareness and communication, (6) motives and willingness to act as well as (7) lack of resources.

Since the costs of virtual conferences are lower (although not necessarily always those of hybrid alternatives) (Puccinelli et al., 2022), the choice of format is more likely to be in the area of willingness and motives. Institutional barriers might play a role, as many conferences are planned by associations whose budget depends on conference fees. Uncertainty about the feasibility of online conferences is also not pertinent as during the pandemic academics improve their videoconferencing skills. Lack of awareness is not relevant since the problem of travel related CO2 emissions is well known among academics. Another reason might be time and possible reaction lags as it can take time to overcome the obstacles of adapting to a new format (Eisenack et al., 2014). However, such reaction lags are not plausible as the pandemic continues over a longer period of time. Wu et al. (2022) suggest that there are logistic problems, time zone issues and an unwillingness to change the status quo.

The limited willingness to organise virtual conferences might be also related to the pro-environmental attitude–behaviour gap which is confirmed for various consumer goods (Kollmuss & Agyeman, 2002; Peattie, 2010) and for travel and tourism behaviour (Higham et al., 2016; Juvan & Dolnicar, 2014). A period of almost two-years of no or only virtual conferences may also have emphasised what aspects cannot be fulfilled by virtual gatherings, such as easy networking, meeting new contacts, time for chit-chatting and possibly also venues with nice amenities and food.

Several robustness checks are performed. First, alternative indicators on restrictions are used including those on public events. The analysis proves to be independent of measures on restrictions. Second, a dummy variable is constructed to determine whether the conference is organised by an Association (for instance, IEEE). However, this variable is not significant at conventional levels (with a t-stat of -0.67 and 0.92, respectively, based on the 38 conferences that mention IEEE in the title). Third, country group dummy variables are included. Since there is a high degree of collinearity between the restriction indicators at the country level and the country dummy variables, this means that only one set of these variables can be estimated at a time. Fourth, estimations based on a sub-sample of conferences planned to be held in Europe are conducted. These results render a much lower probability for virtual conferences than the baseline estimations. Field is also highly significant while the COVID-19 restrictions do not appear relevant. Finally, a multilevel multinomial logit model as suggested by Skrondal and Rabe-Hesketh (2003) is employed where the error term is allowed to vary across country groups, without affecting the main result.

An important assumption underlying the multinomial logit model (MNL) is the property of independence of irrelevant alternatives (IIA). This means that the relative probability of choosing one alternative over another is independent of all other alternatives or are not included in the choice set (Long & Freese, 2014). Because of this, the Hausman and Hsiao tests are performed for the assumption of “independence of irrelevant alternatives" (IIA) for each of the conference modes. Test results demonstrate that the IIA is not violated (*p* > 0.10). The multinomial probit model is also used as an alternative estimation method, where the error terms are assumed to be independent standard normal random variables. These results are similar to those of the multinomial logit model except for a higher significance level of the COVID-19 restriction and the planning time variables (Table 7, Appendix).

## The conclusions

This study contributes evidence on the direction of formats offered for academic conferences in the mature phases of the COVID-19 pandemic. By doing so, factors that influence the choice of format for planned academic conferences are examined, distinguishing between in-person, hybrid and virtual gatherings. Data include 547 calls for papers from organisers in several natural and social science fields published in spring 2022 for academic conferences planned to be held between August 2022 and July 2023. Two thirds of organisers are offering face-to-face conferences in the mature or post-pandemic period, one fifth a hybrid and only 13% opt for a virtual format. Recently discussed conference concepts such as multi-hub conferences are largely ignored.

Estimation results of the multinomial logit model demonstrate that the conference format is largely related to constraints on gatherings in the planned location and to a lesser extent on international travel restrictions in the host country at the time of planning. Another important finding is that not only the proportion of hybrid and virtual conferences is surprisingly low but also tends to fall to almost zero over time. However, there are differences across disciplines: Educational and social sciences are more likely to have virtual conferences, while arts and humanities, natural sciences and computer sciences are least likely to offer the fully online format.

Several conclusions can be drawn from these findings. First, most academic conferences are planned to go back to in-person format, suggesting that organisers (and possibly also the participants) prefer this traditional setup regardless of the improved technology, experience, inclusiveness and environmental gains. This means that the adoption of the technology (innovation) is discontinued, despite the spread throughout its whole society. Possibly, the two-year period of no or only virtual conferences, including elements of video fatigue, makes the benefits of a face-to-face conference crystal clear. It is, however, not unlikely that academics have gained expertise in video meetings for smaller or more administrative events, something that cannot be identified with the dataset at hand and thus is left for future research to investigate. Somewhat surprisingly, the importance of physical meetings also outweighs environmental concerns.

Several implications for stakeholders can be derived from the findings. The new in the mature phase of the Covid-19 pandemic seems to coincide very well with the old way of organising a conference. This means that some of the typical advantages of virtual conferences are lost. Because of this, associations and professional societies may need to consider how to maintain inclusive and environmentally friendly alternatives. One option would be to offer a mix of digital and in-person conferences. Another is the hub-and-spoke model that is not yet widely tested. The main conference could be embedded by certain sessions that are open for online participation, but it is not completely certain what the presumptive participants think of such an arrangement.

There are some limitations of the study that should be noted. First, engineering and technology conferences are not included. Second, several possible conference-specific factors (length, type of conference) are not considered due to data availability. Future work should extend the model and provide a more detailed description of hybrid and online conferences. More variables are also needed, such as the characteristics of conference organisers.

## Appendix

See Tables 6 and 7.

**Table 6:** Restrictions on international travel and gatherings (as of March 2022)

	International travel	Gatherings
Algeria	3	4
Argentina	2	1
Australia	3	4
Austria	1	4
Bahrain	2	2
Belgium	3	3
Brazil	4	4
Bulgaria	1	3
Canada	4	4
Chile	2	2
China	2	4
Congo	1	0
Croatia	2	2
Cyprus	1	2
Czechia	2	3
Denmark	1	0
Ecuador	1	4
Egypt	1	4
Finland	2	0
France	3	2
Georgia	1	4
Germany	3	4
Ghana	1	4
Greece	1	3
Hong Kong	3	4
Hungary	2	2
Iceland	0	0
India	2	4
Indonesia	3	3
Ireland	3	0
Israel	1	0
Italy	2	1
Japan	4	1
Jordan	0	2
Latvia	1	3
Lithuania	1	0
Malaysia	2	4
Mauritius	2	4
Mexico	1	0
Morocco	3	4
Nepal	1	1
Netherlands	3	0
New Zealand	3	3
Nigeria	2	3
Norway	1	0
Peru	1	4
Philippines	3	4
Poland	2	0
Portugal	1	0
Romania	1	2
Russia	3	2
Rwanda	1	1
Serbia	1	2
Singapore	3	4
Slovakia	2	4
Slovenia	0	0
South Africa	1	2
South Korea	2	4
Spain	3	4
Sri Lanka	2	3
Sweden	3	0
Switzerland	3	0
Taiwan	2	0
Thailand	2	3
Tunisia	1	0
Turkey	1	0
UAE	1	1
United Kingdom	1	0
Ukraine	3	3
United States	4	2
Zimbabwe	1	3

Definition of restrictions on gatherings: (0) no restrictions, (1) on very large gatherings (> 1000 persons), (2) on gatherings between, 100 to 1000 persons, (3) restrictions on gatherings between 10–100 persons and (4) restrictions on gatherings of 10 persons or less. Definition of international travel restrictions: (0) no travel restrictions, (1) screening, (2) arrival quarantine from some or all regions, (3) ban on arrivals from certain regions and (4) ban on all regions or total border closure

*Source* Hale et al., (2020, 2021)

**Table 7:** Multinomial probit estimations of conference formats offered

	Hybrid		Virtual	
	Baseline category in-person conference			
	Coeff	z-stat	Coeff	z-stat
*Academic field*				
Arts & Humanities (reference cat. Business & Management)	0.78	1.18	− 1.57**	− 2.50
Educational Science	1.79***	3.03	0.27	0.53
Energy	0.32	0.49	− 1.00*	− 1.80
Health sciences	0.49	0.70	− 1.06**	− 1.99
Mathematics & statistics	0.94	1.60	− 0.46	− 0.87
Social sciences	1.22**	2.08	0.20	0.42
Computer science and IT	0.50	1.03	− 0.91**	− 2.13
Natural science	1.19*	1.77	− 1.03*	− 1.76
*Travel restrictions*				
2 Arrival quarantine from some or all regions (reference: No restrictions/Screening)	0.18	0.76	0.21	0.65
3 Ban on arrivals from some regions	0.12	0.55	0.40	1.25
4 Ban on all regions or total border closure	0.31	1.15	0.95**	2.17
*Restrictions on gatherings*				
2 On gatherings between 100–1000 persons (reference category: very large gatherings/no restrictions)	− 0.02	− 0.06	− 0.01	− 0.04
3 On gatherings between 10–100 persons	0.36	1.33	0.96***	2.61
4 On gatherings of less than 10 persons	− 0.42**	− 2.26	0.19	0.64
Time period starting from May 2022 in months	1.09**	2.12	0.30	0.70
Time period starting from May 2022 in months squared	− 0.07**	− 2.06	− 0.02	− 0.93
Constant	− 5.49***	− 2.83	− 2.07	− 1.18
Log pseudolikelihood	− 444.61			

Standard errors are clustered across countries (#71)

*Source* https://covidtracker.bsg.ox.ac.uk; Easychair and own calculations
